# Transcriptome sequencing reveals key potential long non-coding RNAs related to duration of fertility trait in the uterovaginal junction of egg-laying hens

**DOI:** 10.1038/s41598-018-31301-z

**Published:** 2018-09-04

**Authors:** Adeyinka Abiola Adetula, Lantao Gu, Chinedu Charles Nwafor, Xiaoyong Du, Shuhong Zhao, Shijun Li

**Affiliations:** 1 Key Laboratory of Agricultural Animal Genetics, Breeding, and Reproduction, Ministry of Education, Huazhong Agricultural University, Wuhan, Hubei Province 430070 China; 2 Faculty of Agriculture, Benson Idahosa University, Benin City, Edo State Nigeria; 3 College of Informatics, Huazhong Agricultural University, Wuhan, 430070 China

## Abstract

Duration of fertility, (DF) is an important functional trait in poultry production and lncRNAs have emerged as important regulators of various process including fertility. In this study we applied a genome-guided strategy to reconstruct the uterovaginal junction (UVJ) transcriptome of 14 egg-laying birds with long- and short-DF (n = 7); and sought to uncover key lncRNAs related to duration of fertility traits by RNA-sequencing technology. Examination of RNA-seq data revealed a total of 9977 lncRNAs including 2576 novel lncRNAs. Differential expression (DE) analysis of lncRNA identified 223 lncRNAs differentially expressed between the two groups. DE-lncRNA target genes prediction uncovered over 200 lncRNA target genes and functional enrichment tests predict a potential function of DE-lncRNAs. Gene ontology classification and pathway analysis revealed 8 DE-lncRNAs, with the majority of their target genes enriched in biological functions such as reproductive structure development, developmental process involved in reproduction, response to cytokine, carbohydrate binding, chromatin organization, and immune pathways. Differential expression of lncRNAs and target genes were confirmed by qPCR. Together, these results significantly expand the utility of the UVJ transcriptome and our analysis identification of key lncRNAs and their target genes regulating DF will form the baseline for understanding the molecular functions of lncRNAs regulating DF.

## Introduction

Duration of fertility (DF) is defined as the number of day’s post-fertilisation when viable eggs are produced. It is an important trait in egg-laying hens. For efficient and profitable poultry production prolonged duration of fertility for a breeder stock is vital. Studies on artificial insemination (AI) revealed the relationship between the number of spermatozoa inseminated and embryo survival influence duration of fertility^1^. Prolonged sperm storage and its longevity within the sperm storage tubules (SST) in the uterovaginal junction (UVJ) of laying hens were associated with DF trait^2–4^. The possibility of increasing the AI intervals by improving DF via selection of DF traits has been proven^5–7^.

To date, several proteins and enzyme-coding genes have been associated with fertility potential of laying birds, including, carbonic anhydrase^8^, avidin and avidin-related protein-2 (AVR2)^9^, aquaporins^10,11^, alkaline phosphatase^12^, progesterone receptor^13^, transforming growth factor-β (TGF-β) and its receptors^14^. Additionally, immune system activity has been implicated in maintaining sperm storage within the SST and at the time of fertilization^15,16^. Likewise, breeder and co-workers proposed that energy derived from mitochondrial fatty acid oxidation might be important for sperm longevity, motility, mobility and storage in the oviduct. Despite these extensive studies causes of long-DF, short-DF, and in some cases, infertility in egg-laying hens is largely unknown. Especially the transcriptional and post-transcriptional regulation of gene function during oviductal sperm selection, transport, and storage^17^.

Long non-coding RNAs (lncRNAs) have emerged as important molecules for the transcriptional and post-transcriptional regulation of gene expression evidenced by their tissue-specific expression patterns and subcellular localization^18^. LncRNAs required for spermatogenesis and fertility have been identified in Drosophila^19^. Also, regulatory elements such as enhancers and regulatory non-coding RNAs that are spermatozoa-specific in mammalian species have been identified^20–23^, supporting the possibility that lncRNAs may impact DF in egg-laying hens. In chicken, only a small percentage of all annotated lncRNAs have been functionally characterized^24^, but several lncRNAs have been identified including those associated with skeletal muscle development^25,26^, liver and adipose tissues^27^ as well as several lncRNAs in the male testis with extreme sperm motility^28^.

The sperm storage tubule (SST) located in the uterovaginal junction (UVJ) is considered the main site of the residence of spermatozoa and are highly related to fertility and reproductive traits^29^. Therefore, to identify lncRNAs specifically associated with DF an analytical approach that provides a holistic view of the transcriptional landscape of the UVJ during reproductive phase could shed light on the molecular regulation of DF in egg-laying hens. In this paper, egg-laying hens were divided into two groups; long-DF and short-DF. The long-DF hens have prolonged duration of fertility, large and fertile egg production, while short-DF hens were characterized based on their short duration of fertility, small and infertile egg production. In the present study, targeted analysis of transcriptome was based on RNA isolated from the UVJ of reproductively isolated hens coded as long-and short-DF hens and sequenced using RNA-sequencing technology with the aim of identifying the potential important lncRNAs that are associated with prolonged DF in egg-laying hens. These findings provide some new information for understanding the molecular functions of lncRNAs regulating DF and extend the knowledge of the molecular mechanisms underlying fertility in egg-laying hens.

## Results

### Duration of fertility trait in egg-laying hens

**Figure 1:**
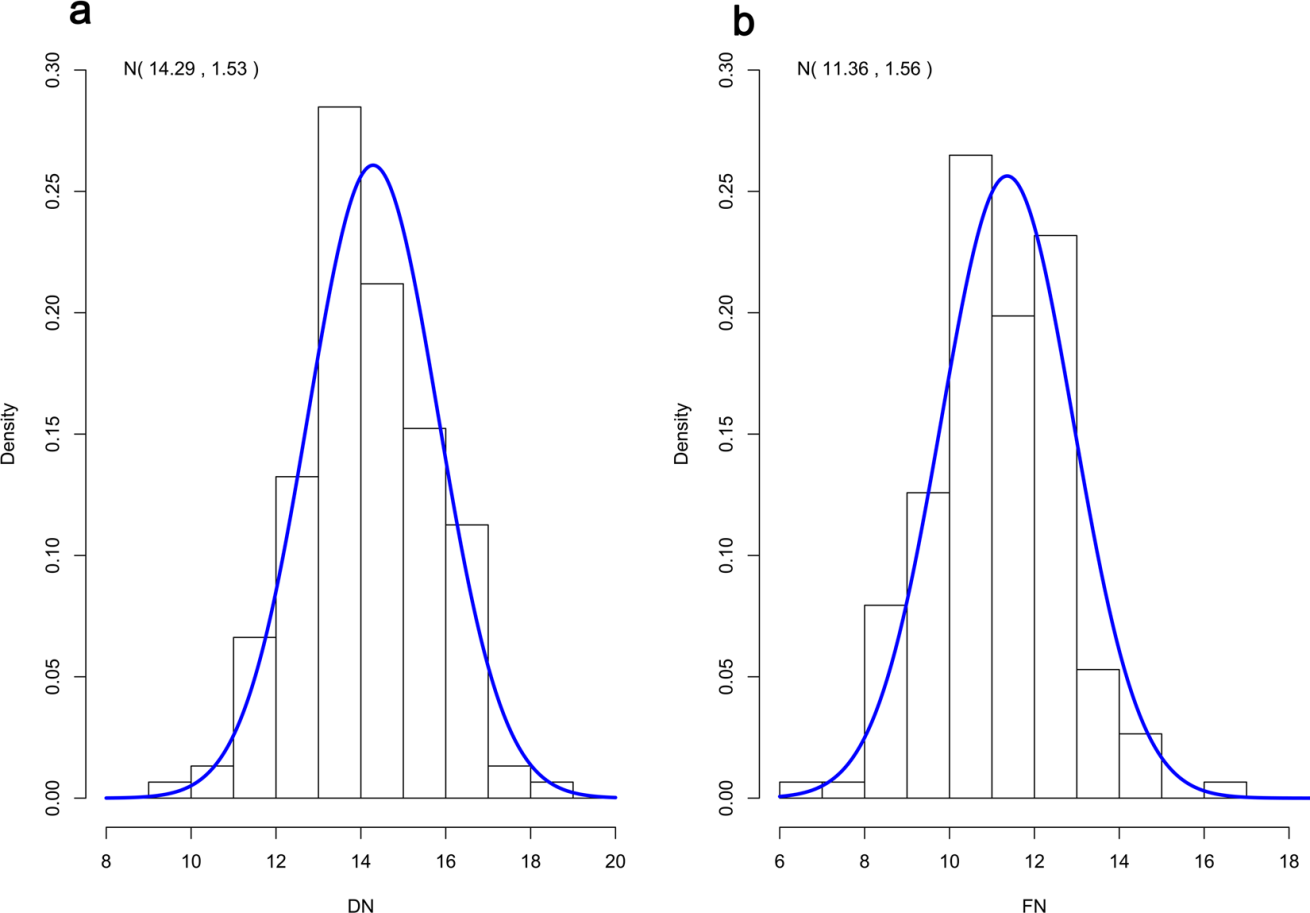
Duration of fertility trait in hens. (**a**,**b**) Histogram illustrates the distribution of DF trait within the population of egg-laying hens (n = 304). DN: the days post-insemination until the last fertile and FN: the numbers of fertile eggs laid after artificial insemination (AI). Bell-shaped indicate the normal distribution and the density curve was determined by standard normal distribution parameters N (µ, σ) where µ is the mean and σ is the standard deviation.

**Figure 2:**
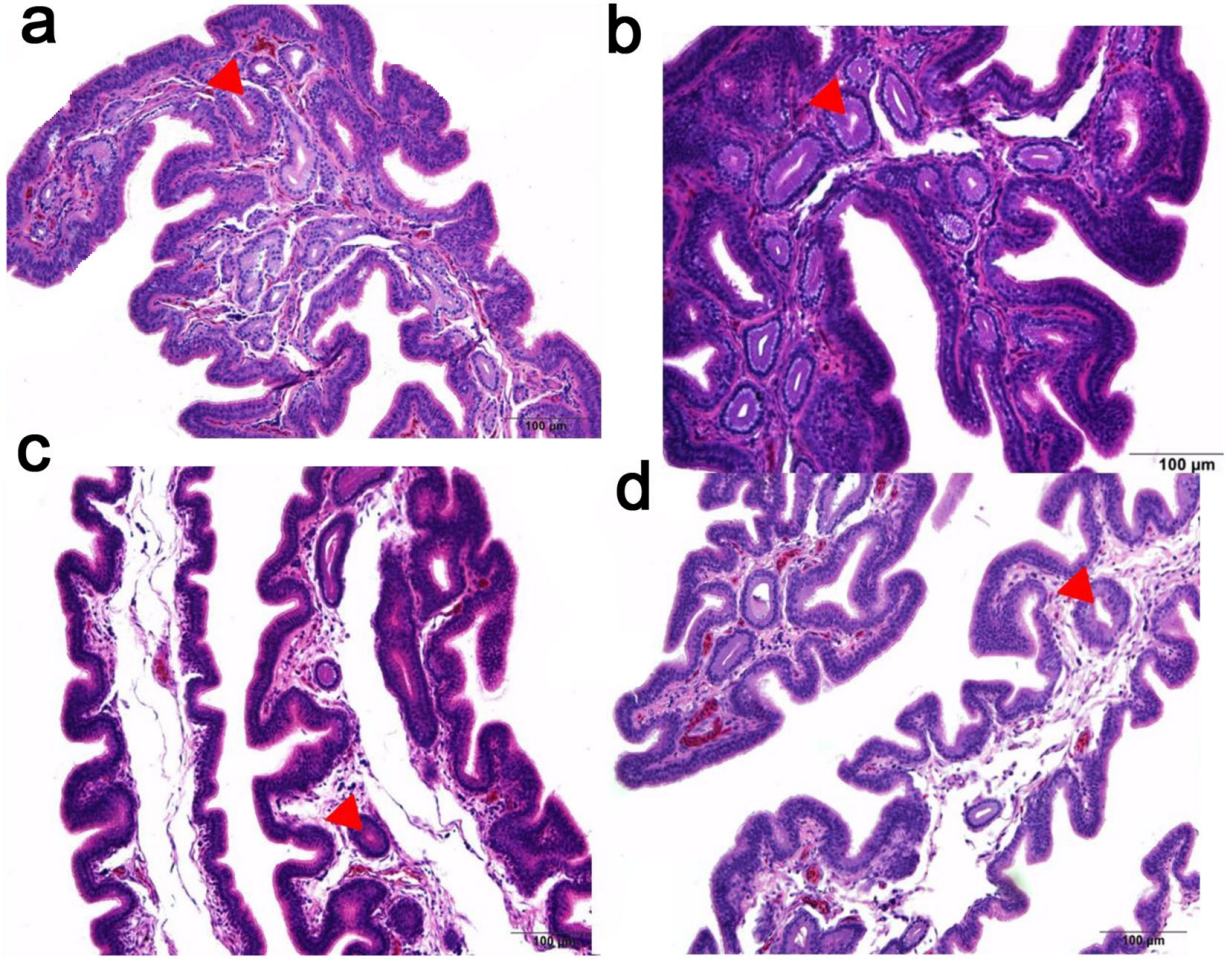
The panel of a tissue section of the UVJ of egg-laying hen. (**a**,**b**) long-DF hens and (**c**,**d**) short-DF hens. A rounded-like shape known as sperm storage tubule (SST) was more embedded in the UVJs of long-DF hens compared with short-DF hens. Data are presented as mean ± SEM (n = 4). P < 0.01 (Student’s t-test). The red arrow indicates the SSTs, Scale bar = 100 μm.

A total of 304 hens were recorded daily by the DF trait: DN: the days post-insemination until the last fertile and FN: the numbers of fertile eggs laid after artificial insemination (AI). In general, there is a wide range of variability in the DF trait. DN ranges between 8 and 19 days and FN ranges between 6 and 17 eggs (Fig. 1). Table 1 showed the characteristics of the DF phenotype in reproductively isolated hens coded as long-and short-DF hens. Both DN and FN showed high individual variability between the two groups even when we observed same and optimal multiple insemination conditions. Furthermore, the ultrastructural analysis of UVJ tissue showed that more SSTs were embedded in the long-DF hens (Fig. 2a,b) compared with short-DF hens (Fig. 2c,d). There was a significant difference in the number of SSTs between the two groups (21.5 ± 4.03 vs. 4.00 ± 0.91) (p < 0.01) in long and short-DF hens respectively. These results were able to distinguish the long and short-DF UVJ tissues.

**Table 1:** Duration of fertility trait in hens.

Phenotypes	DN^*^ (days, Mean ± SD)	FN^†^ (eggs, Mean ± SD)	Fertility rate^‡^ (%)
Long DF	18.71 ± 0.49	17.43 ± 0.53	94.57
Short DF	7.14 ± 1.57	7.43 ± 1.40	23.74

For all phenotypes, we examined multiple inseminations (n = 7). *DN: the days post-insemination until the last fertile egg (up to about 40 wks of age); ^†^FN: the numbers of fertile eggs laid after artificial insemination (AI) (up to about 40 wks of age); ^‡^% fertility = number of fertile eggs/number of total eggs produced or set.

### General characteristics of the egg-laying hen’s transcriptome

**Figure 3:**
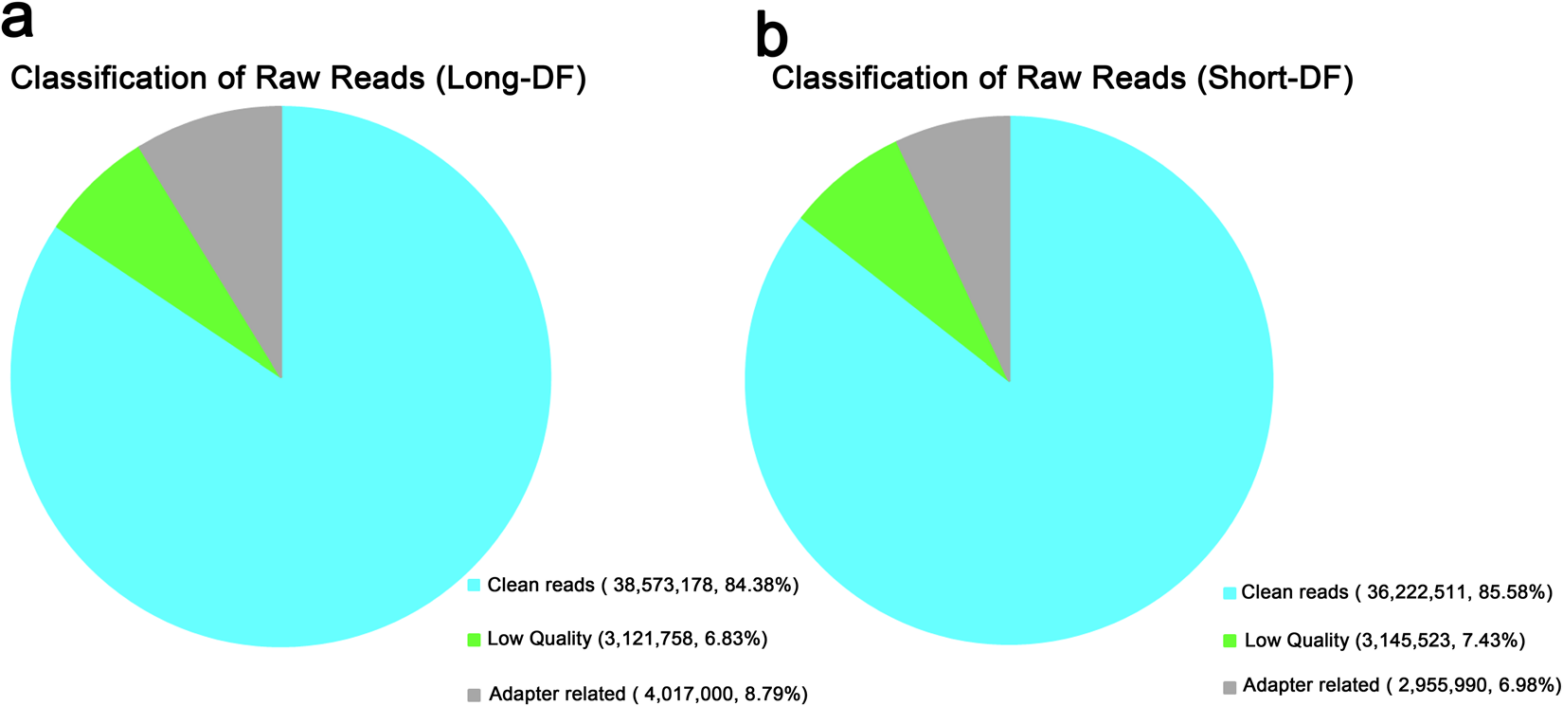
Classification of sequencing raw reads from (**a**) Long-DF and (**b**) Short-DF libraries in hens.

RNA-sequencing was used to analyze the lncRNA expression from 14 cDNA libraries constructed from the UVJ of long- and short-DF hens (no pooled samples, n = 7). On the average, approximately 45.7 and 42.3 million raw reads, were generated from the long-DF and the short-DF groups respectively (Fig. 3). After data pre-processing and quality control, clean reads from the long-DF group ranges from (~80–90%) and short-DF group (~79–92%) were successfully aligned to chicken genome:Galgal4.0, map ratio of reads in most samples was about 73% in both groups; read coverage and mapped unique reads in most samples was more than 70%, see Supplementary Table S1 for the details of data pre-processing and read mapping. The reads alignment to chicken genome indicated that the sequencing reads were of good quality and the sequencing depth was sufficient for further analysis of lncRNA between the two groups of hens.

### Identification of lncRNAs in long-and short-DF egg-laying hens

**Figure 4:**
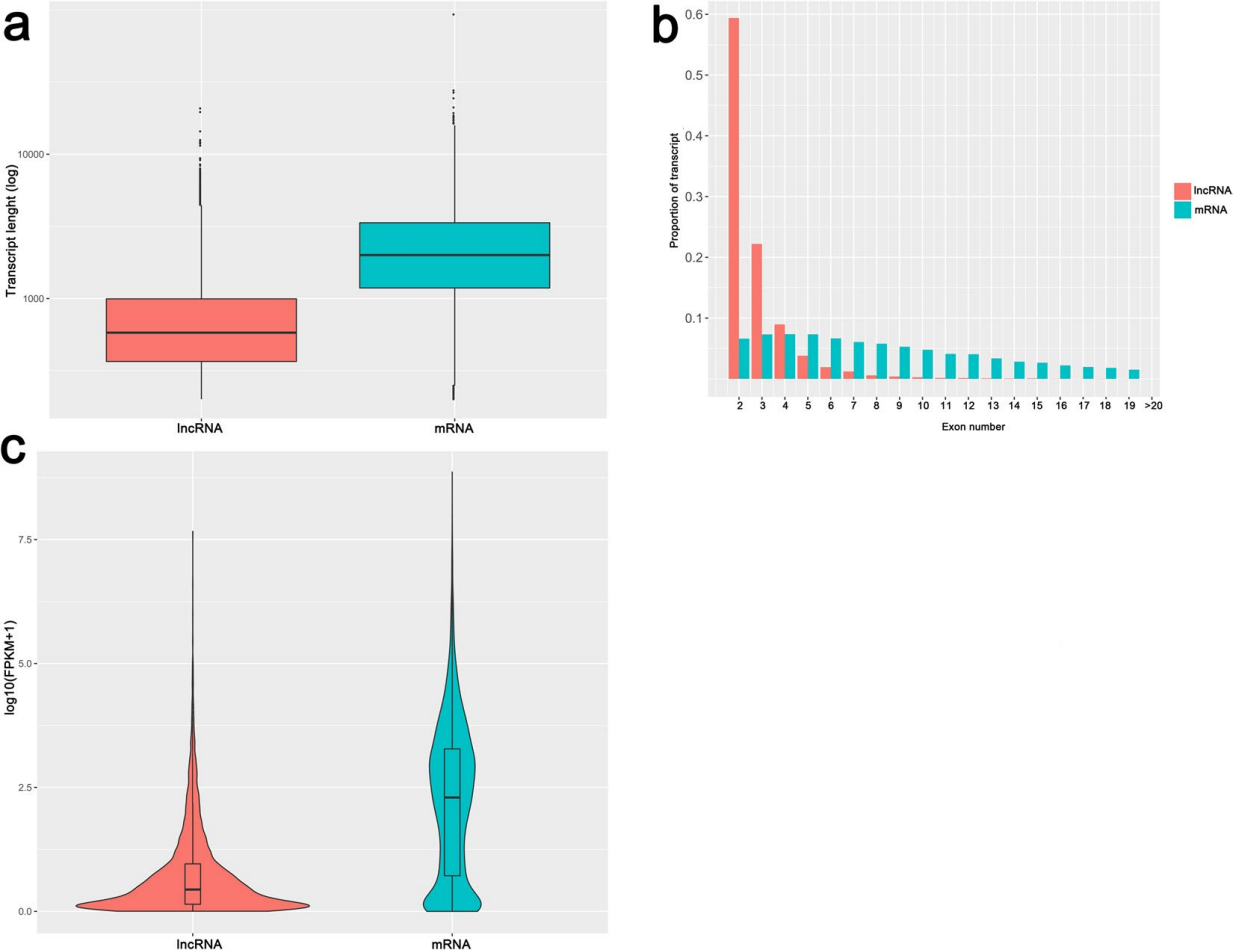
The distribution of transcript length, exon number and expression level of lncRNAs and mRNAs identified in UVJ samples of long-DF and short-DF hens. (**a**) Distribution of identified lncRNA and mRNA transcript lengths. (**b**) Distribution of exon numbers of lncRNA and mRNA transcripts. (**c**) Expression distribution of the lncRNA and mRNA transcripts.

In this study, we adopted a set of filtering criteria as described in the methods to identify putative lncRNAs from the alignments. Consequently, a total of 9977 lncRNAs were discovered in the assembled transcripts for which an average of 7038 and 6909 was expressed lncRNA transcripts in long- and short-DF groups respectively (Supplementary Table S2). Among the total lncRNA transcripts identified, 7401 and 2576 were known and unknown lncRNAs (Supplementary Table S2). Nevertheless, we observed that majority of lncRNAs were from the intergenic region (Supplementary Table S3). Next, the transcript lengths, exon number, and expression level between lncRNAs and mRNAs which generated from 14 individual chicken samples were compared and graphed, as shown in Fig. 4a–c. All the lncRNAs and all the mRNAs were regarded as two groups to compare their basic characteristics. The lncRNA transcript lengths were significantly shorter than those of mRNAs (Fig. 4a). A majority of lncRNAs had two or three exons, whereas mRNAs contained a broad range of exon numbers from two to greater than twenty (Fig. 4b). Additionally, the average expression level detected for lncRNAs (0.74 average FPKM) is significantly lower than that of mRNA (2.2 average FPKM) (Fig. 4c). Together, these results indicated that the lncRNAs in the UVJ of long- and short-DF exhibited relatively short length, low exon numbers, and low expression level.

### Differentially expressed (DE) lncRNAs

**Figure 5:**
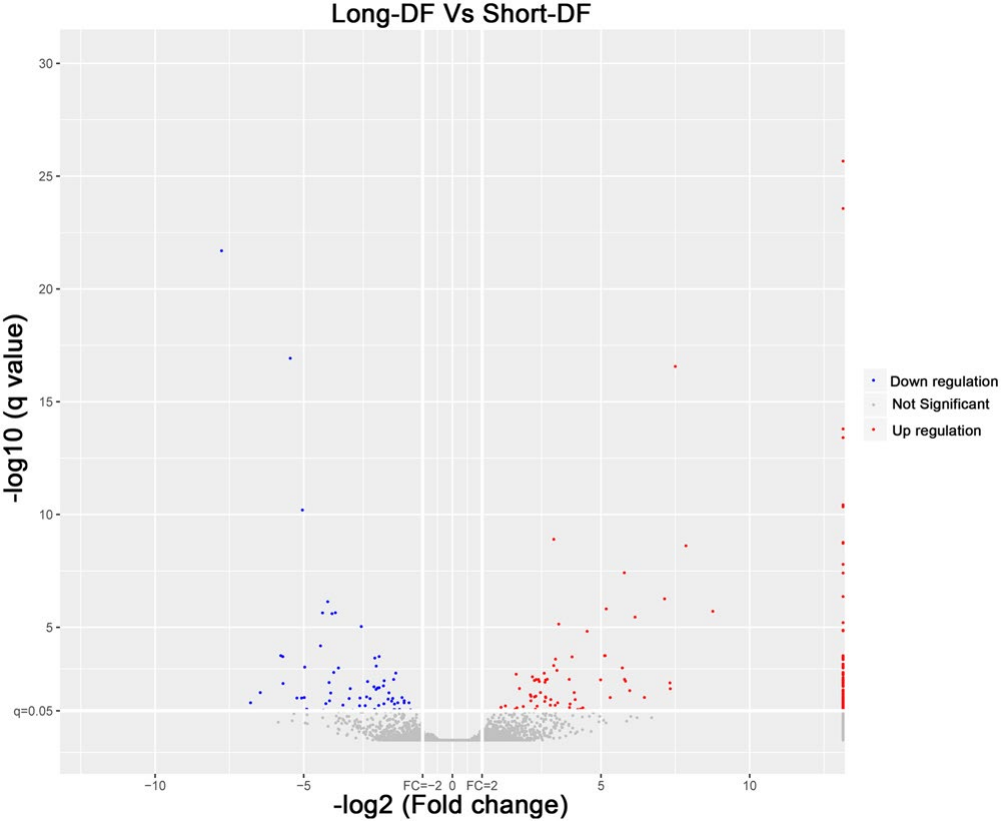
The differentially expressed (DE) lncRNAs between the long- and short-DF hens. The red and blue dots represent up- and down-regulated lncRNAs respectively. The grey dots represent the lncRNAs without significant differential expression.

After identification of the lncRNAs, the DE-lncRNAs were further analyzed based on the criteria of differential expression analysis. In total, the expression levels of 223 lncRNAs were significantly different between the long- and short- DF groups (q value < 0.05). Among these 223 lncRNAs, 81 were up-regulated and 142 were down-regulated in the long-DF group compared with the short-DF group (Fig. 5, Supplementary Table S4). The top 10 up-regulated and down-regulated lncRNAs in the long- DF hens were listed in Table 2. Among the downregulated ones, 85 lncRNAs were located in the intergenic region, 16 lncRNAs in bidirectional, 4 lncRNAs in introns (2 intronic-antisense and 2 intronic-sense) and 37 lncRNAs in exons (34 exonic-antisense and 3 exonic-sense). For the upregulated ones, 55 lncRNAs were mapped in intergenic position, 6 lncRNAs in bidirectional, 15 lncRNAs in exons (12 exonic-antisense and 3 exonic-sense), and 5 lncRNAs in introns (intronic-antisense) (Supplementary Table S4).

**Table 2:** List of top differentially expressed lncRNAs in the long-DF hens identified in the UVJ transcriptome (FDR-corrected P-value < 0.05).

DE-lncRNA ID	lncRNA type	length	log2FC	P value	Q value	Regulation type
MSTRG.2982.4	intergenic	4713	10.55	0.000148	0.032464	UP
MSTRG.11712.1	exonic_antisense	460	9.38	1.7E-06	0.003118	UP
MSTRG.838.2	intergenic	5023	8.95	3.75E-31	1.65E-27	UP
MSTRG.10030.1	intergenic	2695	7.83	1.13E-06	0.000708	UP
MSTRG.1096.2	intergenic	1362	7.59	3.58E-05	0.006568	UP
MSTRG.29422.1	exonic_antisense	5615	7.56	1.79E-20	2.72E-17	UP
MSTRG.7526.1	intronic_antisense	911	7.45	7E-08	4.19E-05	UP
MSTRG.22548.1	intergenic	237	7.39	1.77E-06	0.003118	UP
MSTRG.5381.1	exonic_antisense	344	7.39	1.77E-06	0.003118	UP
MSTRG.6385.5	intergenic	2632	7.01	7.13E-07	0.000305	UP
NONGGAT009046.2	intergenic	2442	−5.93	3.4E-11	4.99E-08	DOWN
MSTRG.26352.3	intergenic	901	−6.04	3.61E-05	0.012891	DOWN
MSTRG.15771.3	intergenic	7399	−6.37	0.000107	0.027697	DOWN
MSTRG.23181.9	exonic_antisense	1229	−6.43	0.000306	0.027473	DOWN
MSTRG.23244.2	intergenic	7559	−6.47	9.55E-05	0.038805	DOWN
MSTRG.15070.6	intergenic	3272	−7.03	2.08E-13	6.09E-10	DOWN
MSTRG.17455.2	intergenic	1861	−7.17	0.00018	0.019517	DOWN
MSTRG.13256.1	intergenic	210	−7.60	8.93E-06	0.011233	DOWN
NONGGAT003930.2	exonic_antisense	1735	−7.64	1.8E-08	1.35E-05	DOWN
MSTRG.2059.3	intergenic	6585	−11.49	1.16E-21	1.04E-17	DOWN

### DE-lncRNAs target genes and Gene Ontology (GO)

**Figure 6:**
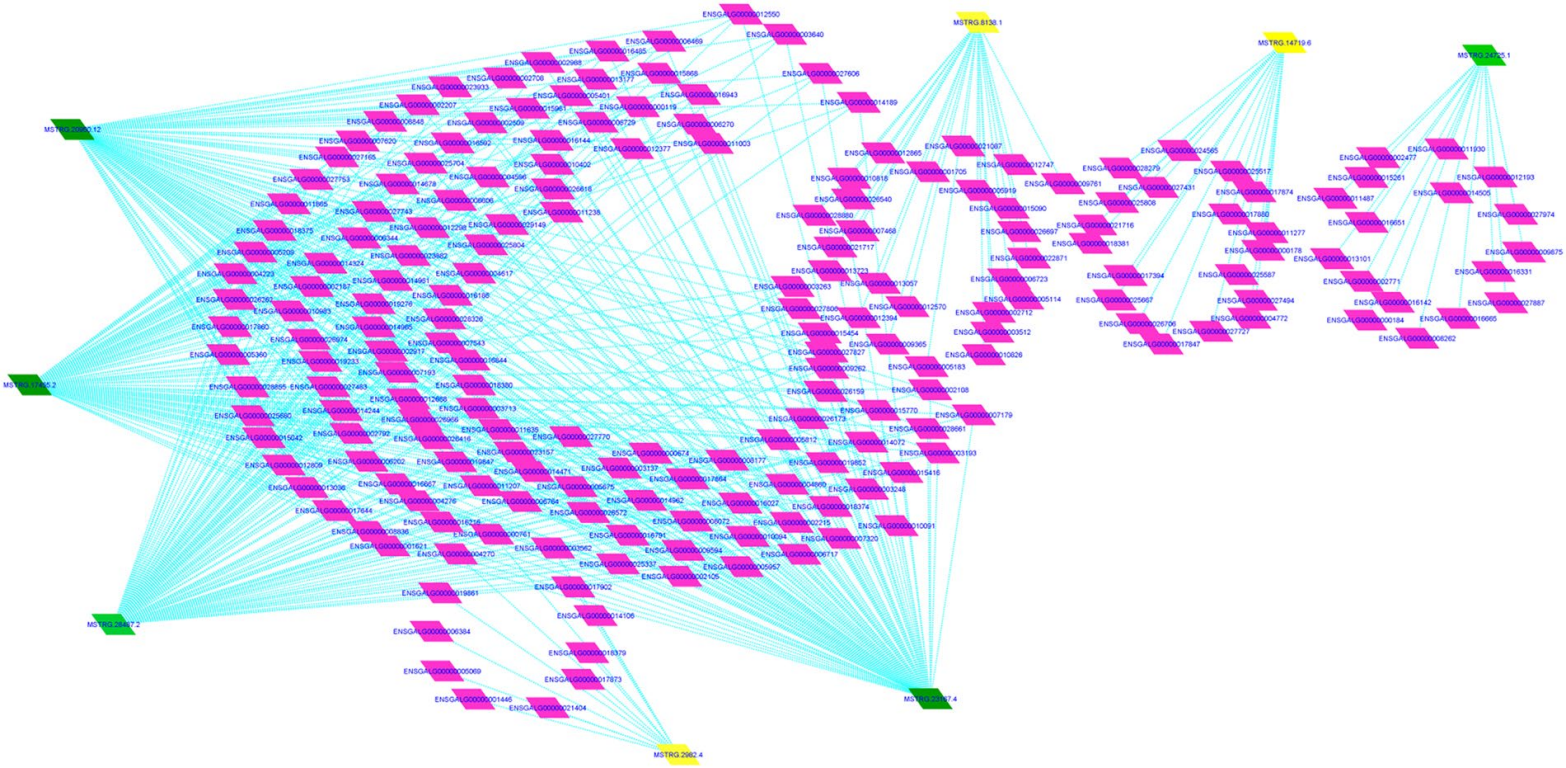
The regulatory network of the 8 differentially expressed lncRNAs involved in regulating the duration of fertility trait in egg-laying hens was constructed. Yellow and green nodes represent upregulated and downregulated lncRNAs in the long-DF group respectively. Pink nodes represent the predicted lncRNA target genes. Lines represent the regulatory relationships between lncRNAs and their potential target genes.

**Figure 7:**
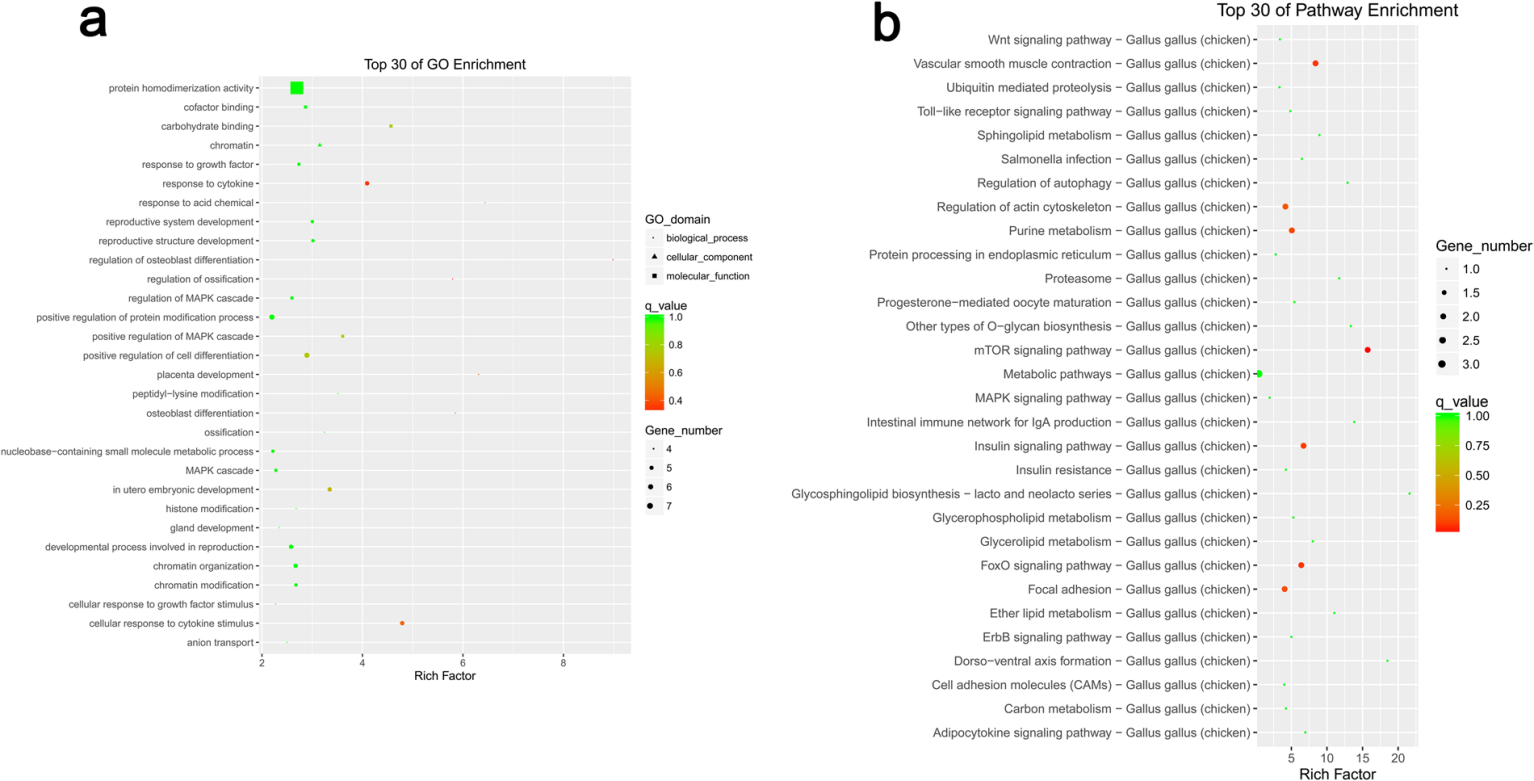
The 30 most enriched functional classifications of DE-lncRNAs target genes between long and short-DF hens. (**a**) The Gene Ontology (GO) of the DE-lncRNAs target genes. The ontology covers three categories: Biological Process, Cellular Component, and Molecular Function. (**b**) The Kyoto Encyclopedia of Genes and Genomes (KEGG) pathway regulated by DE-lncRNAs target genes of long and short-DF hens.

To further investigate the potential biological functions and regulatory relationship label between DE-lncRNAs and target genes, target genes of the DE-lncRNAs were predicted with the Pearson correlation coefficient (PCC) method. Considering PCC > 0.9, and p-value < 0.05, eight DE-lncRNAs between long- and short-DF hens (5 downregulated and 3 upregulated ones) were identified and targeted more than 200 genes. The regulatory network of 8 DE-lncRNAs consisted 200 genes and 530 regulatory relationships (Supplementary Table S5). These results demonstrate a strong regulatory relationship between the 8 DE-lncRNAs and targets in DF trait regulation (Fig. 6). Among the 8 DE-lncRNAs, downregulated *MSTRG.20950.12*, *MSTRG.17455.2*, *MSTRG.23167.4*, and MSTRG.28487.2 targeted the majority of genes, such as *SOX2*, *FAM20C*, *EVPL*, *PHGDH*, and *PRPS2*. Upregulated *MSTRG.14719.6* targeted set of genes encoding small nucleolar RNAs, C/D box (SNORD) and small nucleolar RNAs, H/ACA box (SNORA), such as *SNORD38*, *SNORD14*, *SNORA32*, *SNORA77*, *SNORA66*, *SNORA55*, and *SNORA14*. MSTRG.8138.1 regulated genes like *CYP51A1* and *17.5*. MSTRG.2982.4 also negatively targeted genes such as *IFIT5*, and MSTRG.24725.1 positively regulate genes like HMGI-C (Supplementary Table S5). The most represented target gene (*SOX2*) of *MSTRG.20950.12*, *MSTRG.17455.2*, *MSTRG.23167.4*, *MSTRG.28487.2*, *MSTRG.14719.6*, *MSTRG.2982.4*, *MSTRG.24725.1* and *MSTRG.8138.1* were enriched in biological function such as reproductive structure development, reproductive system development, positive regulation of cell differentiation, and response to growth factor (Fig. 7a, Supplementary Table S6). In addition, the target genes of downregulated lncRNA (*MSTRG.20950.12*) such as *CTR9* and *DDIAS* were enriched in the GO functions like response to cytokine, positive regulation of MAPK cascade, chromatin, and developmental process involved in reproduction (Supplementary Table S6). Furthermore, according to pathway enrichment analyses, the target genes of *MSTRG.8138.1* (e.g., *BRAF*, and *PRKAA2*) were significantly enriched in the KEGG functions like mTOR signaling pathway (Fig. 7b, Supplementary Table S7). Together these results showed that these 8 key DE-lncRNAs may regulate DF trait, UVJ formation, and reproductive processes through their potential targets (Table 3).

**Table 3:** Summary of the eight key differentially expressed long non-coding RNA (lncRNA) associated with duration of fertility trait in the egg-laying hens.

The bold genes are the overlapped lncRNA targets in the GO and KEGG pathways.			
DE-LncRNA	Category	Function	Targets
MSTRG.23167.4	MF	Identical protein binding	AADAT, TOX3, VWF, CEBPB, HNF1A, SHMT1,**CLDN1**, ACPP
MSTRG.28487.2, MSTRG.17455.2, MSTRG.8138.1, MSTRG.2982.4	BP	Response to growth factor	**SOX2**, NOS1, **FAM20C**, BMPER, NPNT
MSTRG.28487.2, MSTRG.8138.1	BP	Reproductive system development	**SOX2**, BIRC6, HNF1A,VWF, CEBPB,
MSTRG.14719.6, MSTRG.28487.2, MSTRG.17455.2, MSTRG.8138.1, MSTRG.2982.4	BP	Positive regulation of cell differentiation	**SOX2**, BNIP2, ZNF335, **FAM20C**, CEBPB, NPNT, **PLCB1**
MSTRG.20950.12, MSTRG.14719.6, MSTRG.24725.1	BP	Cellular response to cytokine stimulus	**CTR9, PLCB1, DDIAS**, NPNT, PID1, FASN
MSTRG.24725.1, MSTRG.28487.2, MSTRG.8138.1	BP	Developmental process involved in reproduction	VWF, CEBPB, BIRC6, HNF1A, **DDIAS, SOX2**,
MSTRG.28487.2, MSTRG.17455.2, MSTRG.8138.1, MSTRG.2982.4	BP	Regulation of osteoblast differentiation	NPNT, CEBPB, **SOX2**, **FAM20C**,
MSTRG.28487.2, MSTRG.8138.1	BP	Response to acid chemical	LGMN, **SOX2**, PID1,CEBPB
MSTRG.28487.2, MSTRG.17455.2, MSTRG.8138.1, MSTRG.2982.4	BP	Osteoblast differentiation	**FAM20C**, **SOX2**, NPNT,CEBPB
MSTRG.8138.1	MF	Carbohydrate-binding	**17.5**, CD93
MSTRG.20950.12, MSTRG.14719.6, MSTRG.24725.1	BP	Response to cytokine	**CTR9**, FASN, NPNT, PID1, **DDIAS, PLCB1**
MSTRG.14719.6, MSTRG.14719.6, MSTRG.28487.2, MSTRG.8138.1	BP	Positive regulation of MAPK cascade	**PLCB1**, NPNT, BMPER, BNIP2, **SOX2**
MSTRG.20950.12	BP	Peptidyl-lysine modification	NOS1,YEATS2, ZNF335, **CTR9**
MSTRG.20950.12, MSTRG.14719.6	CC	Chromatin	**CTR9**, CEBPB, **PLCB1**, H3F3B
MSTRG.20950.12	BP	Chromatin organization	ZNF335, **CTR9**, H3F3B,YEATS2, NOS1, HNF1A
MSTRG.20950.12	BP	Chromatin modification	HNF1A, NOS1,**CTR9**, ZNF335,YEATS2
MSTRG.20950.12	BP	Histone modification	YEATS2, NOS1,**CTR9**, ZNF335
MSTRG.14719.6, MSTRG.24725.1, MSTRG.28487.2, MSTRG.8138.1	BP	Regulation of MAPK cascade	BNIP2, **SOX2**, BMPER, NPNT, **PLCB1**
MSTRG.14719.6, MSTRG.24725.1, MSTRG.28487.2, MSTRG.8138.1	BP	MAPK cascade	BMPER, NPNT, **PLCB1, SOX2**, BNIP2
MSTRG.14719.6, MSTRG.24725.1, MSTRG.28487.2, MSTRG.8138.1	BP	Positive regulation of protein modification process	NOS1, **SOX2**, BNIP2, NPNT, **PLCB1**, **CTR9**, BMPER
MSTRG.14719.6, MSTRG.24725.1, MSTRG.28487.2, MSTRG.8138.1	BP	Regulation of cell cycle	PID1, E2F5, **PLCB1, DDIAS**, BIRC6, **SOX2**
MSTRG.28487.2, MSTRG.17455.2, MSTRG.8138.1, MSTRG.2982.4	BP	Ossification	NPNT, CEBPB, **FAM20C**, **SOX2**
MSTRG.8138.1	KEGG	mTOR signaling pathway	**PRKAA2, BRAF**

### Validation of DE-lncRNAs and target genes by qPCR

**Figure 8:**
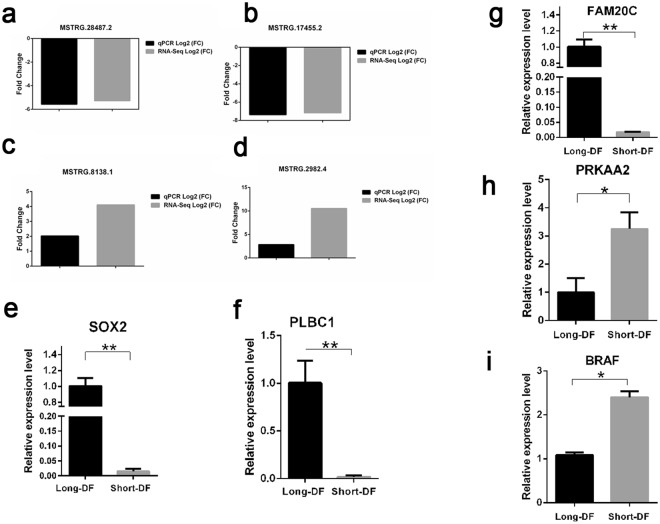
Validation of differentially expressed lncRNAs by qPCR. (**a**,**b**) Fold change expression of down-regulated lncRNAs in long DF hens compared with the short DF hens; (**c**,**d**) Fold change expression of up-regulated lncRNAs in long DF hens compared with short DF hens. Data are reported for three technical replicates in all biological samples. (**e**–**i**) The expression levels of lncRNA target genes between long and short DF hens. Each histogram represents the level of the target gene relative expression level. Error bars indicate standard error of the mean (SEM). Asterisks symbolized P-value significance *p ≤ 0.05, and **p ≤ 0.01, based on assuming unequal variances Student’s t-test.

A total of 4 DE-lncRNAs and 5 target genes were selected and validated by qPCR including two downregulated lncRNAs (*MSTRG.28487.2*, and *MSTRG.17455.2*), two upregulated lncRNAs (*MSTRG.8138.1* and *MSTRG.2982.4*) and five target genes (*SOX2*, *FAM20C*, *PLCB1*, *BRAF* and *PRKAA2*) between the long and short- DF hens. Among the selected genes, downregulated *MSTRG.28487.2* and *MSTRG.17455.2* expression fold change by qPCR were similar to the expression fold change obtained from the RNA-Seq (Fig. 8a,b). Similarly, upregulated *MSTRG.8138.1* and *MSTRG.2982.4* also appeared to be expressed at the same level when compared with the RNA-Seq data (Fig. 8c,d). Together, these data indicated that there was a strong agreement between the qPCR and RNA sequenced data. We found that target genes (*SOX2*, *PLCB1*, and *FAM20C*) were highly expressed in long-DF hens compared with the short-DF hens (Fig. 8e–g). High expression of *BRAF* and *PRKAA2* were also detected in the short-DF hens compared with the long-DF hens (Fig. 8h,i). According to these findings, long- and short-DF hens significantly differed at P < 0.01 and P < 0.05 levels based on assuming unequal variances Student’s t-test.

## Discussion

Duration of fertility (DF) trait is a vital factor in poultry production. In the current study, the estimated DF-trait for the two composite characteristics (DN and FN) was approximately (18.71 ± 0.49; 7.14 ± 1.57) and (17.43 ± 0.53; 7.43 ± 1.40) in the long- and short-DF hens respectively (Table 1). During the reproductive phase, there was a significant difference in the DF traits between the two groups (Supplementary Table S8). In addition, a different morphological change was observed in their respective UVJ samples. Numerous SSTs were embedded in the long-DF groups compared with the short-DF, indicating that the mechanism regulating prolonged DF could be mostly due to the observed SST as revealed in the previous work conducted in broiler and turkey hens^30^. Several studies associated with period of fertility of egg-laying hens have been reported^31,32^. However, the molecular mechanisms underlying prolonged-DF remain obscure.

During the reproductive season, we compared the lncRNA expression profiles in the long-DF hens with those of short-DF hens and identified a set of lncRNA genes associated with DF trait (Supplementary Table S2). In agreement with previous reports^28^, lncRNAs shared similar features, such as shorter in length, lower in exon number, and lower expression level than protein-coding transcripts. A total of 223 lncRNAs were differentially expressed (DE), of these, 81 lncRNAs were upregulated and 142 lncRNAs were downregulated in the long-DF groups, indicating that the majority of the DE-lncRNAs were downregulated throughout the reproductive phase. We anticipate that some of these DE-lncRNAs might play key roles in prolonging the DF in hens as shown in other studies^19,33^.

The DE-lncRNA-target prediction and functional interaction network revealed eight key DE-lncRNAs related to long-DF hens, including *MSTRG.20950.12*, *MSTRG.17455.2*, *MSTRG.23167.4*, *MSTRG.28487.2*, *MSTRG.14719.6*, *MSTRG.2982.4*, *MSTRG.24725.1*, and *MSTRG.8138.1* (Supplementary Table S5). Many of the long-DF-enriched target genes were categorized into identical protein binding, response to growth factor, reproductive system development, positive regulation of cell differentiation, cellular response to cytokine stimulus, developmental process involved in reproduction, regulation of osteoblast differentiation, response to acid chemical, osteoblast differentiation, carbohydrate binding, response to cytokine, positive regulation of MAPK cascade, peptidyl-lysine modification, chromatin, chromatin organization, chromatin modification, histone modification, regulation of MAPK cascade, MAPK cascade, positive regulation of protein modification process, regulation of cell cycle, and ossification.

A total of nine overlapped targets were potentially associated with long-DF hens (Table 3). These included *CLDN1*, *SOX2*, *FAM20C*, *PLCB1*, *CTR9*, *DDIAS*, *17.5*, *PRKAA2*, and *BRAF*. For instance, *SOX2* was expressed at high levels in the long-DF hens compared with the short-DF hens. *SOX2* is a member of the sex-determining region (SRY-related) having analogous HMG domains for DNA binding and is highly involved in regulation of cell differentiation^34,35^. *SOX2* expression patterns in early chicken embryos, consistently mark neural primordial cells at various stages of development^36^. Mutations in *SOX2* have previously been associated with male genital tract abnormalities^37^. Another interesting gene, named family with sequence similarity 20, member C (*FAM20C*), was also highly expressed in the long-DF hens. *FAM20C* is a physiological Golgi casein kinase that phosphorylates multiple secreted proteins^38^. The molecular activity of *FAM20C* has been implicated in mammalian reproduction^39^. In this study, the observation of significant high expression of *FAM20C* implied that it may contribute to the DF regulation of long-DF hens. The immunological response in the UVJ of mated hens requires up-regulated phospholipase C eta 1 (*PLCH1*), an immune-modulatory gene in the previous studies^40^. Likewise in our data, the high expression level of phospholipase C beta 1 (*PLCB1*) was observed in the long-DF hens which suggest that *PLCB1* has a potential role in DF regulation^41^.

One important signaling pathway associated with DF trait is mTOR signaling pathway and the target genes involved were over-represented in KEGG database, including mTOR signaling pathway, Vascular smooth muscle contraction, insulin signaling pathway, FoxO signaling pathway, Regulation of actin cytoskeleton, and Focal adhesion pathway (Supplementary Table S7). The elevated expression of *BRAF* and *PRKAA2* genes via mTOR signaling pathway was seen in the short-DF hens. The mTOR signaling pathways are critical regulators of ovarian function including quiescence, activation, and survival of primordial follicles (PFs), granulosa cell (GC), proliferation and differentiation, and meiotic maturation of oocytes^42^. mTOR signaling pathway has been implicated in fertile egg production and female fertility in mice^43^, suggesting that *BRAF* and *PRKAA2* genes via mTOR signaling pathways may be involved in regulating the DF trait.

In the lncRNA-target regulatory network, several set of genes encoding small nucleolar RNAs, such as *SNORD38*, *SNORD14*, *SNORA32*, *SNORA77*, *SNORA66*, *SNORA55*, and *SNORA14* also exhibited a strong correlation with the upregulated *MSTRG.14719.6*, suggesting the important role of *MSTRG.14719.6* in the post-transcriptional modification of ribosomal RNAs (rRNAs) which are essential for sperm viability and male fertility^44,45^. Additional studies are needed to determine the precise function of this small nucleolar RNAs in the DF regulation.

Furthermore, in the regulatory network, *MSTRG.2982.4* and *MSTRG.24725.1* targeted cytokines related genes such as Mx (Interferon-induced GTP-binding protein Mx) and IFIT5 respectively. Previous studies have that ovarian functions are regulated by cytokines^46–48^, we speculate *MSTRG.2982.4* and *MSTRG.24725.1* may participate in prolonging the DF, likely via their potential target genes. *MSTRG.8138.1* regulated lectin-like proteins (17.5). It has been reported that the sperm-oocyte binding in the mouse occurred as a result of the existence of lectin-like proteins on the sperm plasma membrane which binds to zona pellucida glycoproteins^49^. *MSTRG.8138.1* also played an important role in gene-gene networks that are involved in reproductive system development and positive regulation of cell differentiation, suggesting the crucial roles of *MSTRG.8138.1* in DF regulation. The enrichment of *MSTRG.20950.12* target in cellular response to cytokine stimulus indicates that it may contribute to the immune function within the oviduct of hens during reproductive seasons^50,51^. In addition to these genes, several genes interacted with another in the gene pathways. Of those, 20 additional genes were found to have functions for DF trait regulation, such as *AADAT*, *TOX3*, *VWF*, *CEBPB*, *SHMT1*, *ACPP*, *NOS1*, *BMPER*, *NPNT*, *BNIP2*, *ZNF335*, *BIRC6*, *HNF1A*, *PID1*, *FASN*, *LGMN*, *CD93*, *YEATS2*, *H3F3B*, and *E2F5*.

## Conclusions

In summary, based on the RNA-Seq and bioinformatics analysis results, a list of DE-lncRNAs related to the cellular response to cytokine, reproductive structure development, regulation of protein modification, carbohydrate binding, co-factor binding, chromatin organization and modification, response to growth factors, cytokine stimulus and immune pathways were identified in the UVJ of long and short-DF hens. Eight key DE-lncRNAs were identified to have the most probable role in prolonging the DF in egg-laying hens, thus, they are novel lncRNAs that are related to DF in hens. These results provided the starting point for studies aimed at understanding the molecular mechanism of the DF trait in egg-type chickens.

## Material and Methods

### Egg-laying hens

A total of 304 egg-type chickens obtained from the poultry farm of Huadu Yukou Poultry Industry Co. Ltd, Beijing, China were raised in individual cages, kept in identical light/dark cycles and fed standard diets ad libitum from 25 weeks until the end of the experiment aiming at study their duration of fertility. All hens were artificially inseminated once with 2 × 10^8^ pooled sperms ejaculates collected from viable rooster flocks. Eggs were collected and marked daily from day 2–20 after artificial insemination, all experiment was executed in three replicates and lasted 60 days. The number of egg per hen over the period was recorded and the fertilized eggs were examined by candling on day 10 of incubation (dead embryos were considered as fertile). DF trait was expressed in terms of DN (the number of days post-insemination until last fertile egg) and FN (the number of fertile eggs after a single AI) and hens with DN or FN traits ≥ 18 were selected, coded as long and DN or FN < 10 as short-DF groups^52–55^. Seven long-DF and seven short-DF hens were selected for sampling UVJ. All selected hens were anesthetized with sodium pentobarbital (20 mg/kg) administered intraperitoneally, and the narrow band known as UVJ located at the cranial anterior end of the vagina were used as a sample for RNA collection^29^. In other to characterize the UVJ structure of long- and short-DF hens. The sample size for tissues analysis was four UVJs selected from 4 different individuals of long- and short-DF groups respectively. The UVJ samples were formalin-fixed for 48 h and paraffin-embedded. The 100 μ m of the tissues sectioned were stained with hematoxylin and counterstained with eosin (hematoxylin for 1 min and 1% eosin for 10 sec) for observation under a light microscope (OLYMPUS; TH4-200; Tokyo, Japan).

### RNA isolation and sequencing procedures

Total RNA was isolated from the UVJ of seven long-DF and seven short-DF hens using the RNeasy mini kit (Qiagen, Germany). The quality and concentration of RNA were analyzed by NanoDrop ND2000 (Thermo Fisher Scientific, Waltham, MA, USA) spectrophotometer and gel electrophoresis. Fourteen cDNA libraries were constructed using the TruSeq® Stranded Total RNA Sample Preparation kit (Illumina, USA). The library construction was performed by a commercial company (Shanghai Biotechnology Corporation (SBC), China. The datasets (14 files) supporting the conclusions of this article is available in the Gene Expression Omnibus (GEO) database NCBI under the accession number GSE101163 (https://www.ncbi.nlm.nih.gov/geo/query/acc.cgi?acc=GSE101163).

### Transcriptome analysis for lncRNA data

Mapping of all clean reads was carried on the chicken reference genome (assembly Gallus gallus_4.0) using TopHat (version:2.0.9) with parameters set to default values^56^. Unmapped reads were trimmed and remapped, additionally, Gene Transfer Format (GTF) of the Ensembl gene annotation was included in the read mapping as previously described by^57^. Next, RNA-Seq alignments for all sample was assembled using String Tie (version: 1.3.0) and Gffcompare (version: 0.9.8) was used to evaluate the accuracy of the assembled transcripts (i.e., putative transcripts containing both coding and noncoding transcripts) and compare the assembly with known transcripts (putative transcripts for each sample assembly against a set of combined gene annotations), to extract assemblies that fully match known and unknown annotations^57,58^. Prior to that, low-quality assemblies were removed based on the Fragments Per Kilobase of exon per Million fragments mapped (FPKM) threshold, and multi-exon transcripts were retained for downstream processing. The FPKM threshold for classifying complete and partial transcripts in our experiment was established according to^57^. Furthermore, transcripts with short read length <200 bp, <2 exons including those with maximum putative ORFs < 300 bp were filtered out. Next, protein-coding transcripts were identified and removed with the software programme Coding-Non-Coding Index (CNCI > 0)^57^ and Coding Potential Calculator (CPC > 0)^59^. Similarly, Pfam (http://pfam.xfam.org) was used to remove transcripts with significant protein domain. From the remaining transcripts, a putative lncRNA is defined as novel lncRNAs if its Pfam hits returned insignificantly. The analysis protocols are described step by step in Supplementary Fig. S1. Afterward, the novel lncRNAs were classified according to^60^ and characterized based on transcription length, exon number and expression level between lncRNA and mRNA transcripts (RefSeq transcripts, NCBI). Furthermore, differentially expressed lncRNAs (DE-lncRNAs) in the comparison groups of long-DF versus short-DF were identified using the EdgeR package (freely available from the Bioconductor web site (http://bioconductor.org)^61^. Only the lncRNAs with the criteria of log2FC (fold change) ≥2 and p-value ≤ 0.01 and FDR ≤ 0.05 were identified as DE-lncRNAs.

### Prediction and functional annotation of DE-lncRNAs target genes

To further predict the DE-lncRNA target genes, the Pearson correlation coefficient (PCC) was calculated to evaluate the co-expression relationships between DE-lncRNAs and target genes. The co-expression pairs with PCC > 0.9 and p-value < 0.05 were selected for the construction of the regulatory network, which was visualized by Cytoscape 3.3.0 (free download: available at http://www.cytoscape.org/). Functional enrichment analysis and pathway analysis of DE-lncRNAs target genes were analyzed using Go (Gene Ontology) function in the Database for Annotation, Visualization and Integrated Discovery (DAVID, from the National Institute of Allergy and Infectious Diseases (NIAID), NIH (https://david.ncifcrf.gov/). and Kyoto Encyclopedia of Genes and Genomes (KEGG) (http://www.genome.ad.jp/kegg/) databases^62,63^.

### Quantitative real-time PCR (qPCR) validation of DE-lncRNAs and target genes

The RNA sequencing data were validated by qPCR, using a standard EasyScript™ one-step gDNA Removal, cDNA Synthesis SuperMix, for cDNA production and SYBR Green real-time PCR (Toyobo co., Ltd, Osaka, Japan) following manufacturer’s instructions. Two up-regulated and two down-regulated DE-lncRNAs and five target genes were randomly selected to quantify the expression levels. Quantitative PCR (qPCR) was then performed in Bio-Rad thermal cycler, CFX-384, real-time system. Gene expression in each sample was normalized to beta-actin expression. The qPCR amplifications were conducted using an independent set of seven biological replicates and three technical replicates per sample. Relative quantitation of lncRNAs and target genes expression was evaluated by the 2(−ΔΔCt) methods^64,65^. Primers sequences of lncRNAs and target genes for qPCR are listed in Supplementary Table S9.

### Ethics approval and consent to participate

The dissection of the UVJ sample and collection was conducted according to the guidelines established for the Care and Use of Laboratory Animals by the Ethics Committee of Huazhong Agricultural University, P. R. China, and Standing Committee of Hubei People’s Congress (No. 5) approved by the Animal Care Committee of Hubei Province, P.R. China.

## Electronic supplementary material

Supplementary Dataset 1

Supplementary Dataset 2

Supplementary Dataset 3

Supplementary Dataset 4

Supplementary Dataset 5

Supplementary Dataset 6

Supplementary Dataset 7

Supplementary Dataset 8

Supplementary Dataset 9

Supplementary Figure S1

## Data Availability

All replicates sequencing reads used in this study have been submitted to Gene Expression Omnibus (GEO) under the accession number GSE101163.
