# Supplementary material for: Transcriptome sequencing reveals key potential long non-coding RNAs related to duration of fertility trait in the uterovaginal junction of egg-laying hens
Source: Sci Rep. 2018 Sep 4;8:13185. doi: 10.1038/s41598-018-31301-z (PMC6123486; doi:10.1038/s41598-018-31301-z)
Supplement: Supplementary file 10 — Supplementary Figure S1 [file 41598_2018_31301_MOESM10_ESM.pdf]

Transcriptome sequencing reveals key potential long non-coding RNAs related to duration of fertility trait in the uterovaginal junction of egg-laying hens

Adeyinka Abiola Adetula, Lantao Gu, Chinedu Charles Nwafor, Xiaoyong Du, Shuhong Zhao and Shijun Li

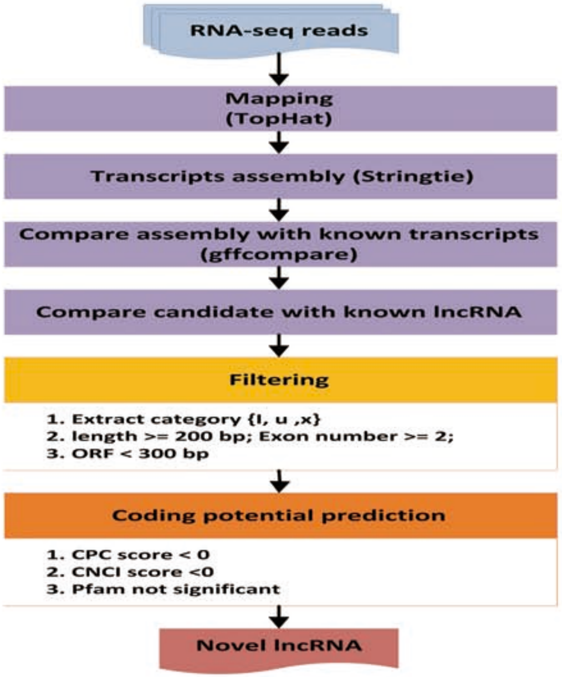

Supplementary Figure S1 The rigorous analysis pipeline used for this study, i, u, and x represent Transfrag falling entirely within a reference intron, Unknown intergenic transcript, and Exonic overlap with reference on the opposite strand respectively.
